# The potential and pitfalls of narrative elicitation in person‐centred care

**DOI:** 10.1111/hex.12998

**Published:** 2019-11-19

**Authors:** Öncel Naldemirci, Nicky Britten, Helen Lloyd, Axel Wolf

**Affiliations:** ^1^ Department of Social Work Umeå University Umeå Sweden; ^2^ University of Exeter College of Medicine and Health St Luke's Campus Exeter UK; ^3^ School of Psychology University of Plymouth Plymouth UK; ^4^ Institute of Health and Care Sciences Sahlgrenska Academy University of Gothenburg Gothenburg Sweden; ^5^ Centre for Person‐Centred Care (GPCC) University of Gothenburg Gothenburg Sweden

**Keywords:** admission interview, lifeworld goals, narrative elicitation, person‐centred care

## Abstract

**Background:**

Revitalized interest in narrative has informed some recent models of patient and person‐centred care. Yet, scarce attention has been paid to how narrative elicitation is actually used in person‐centred care practice and in which ways it is incorporated into clinical routine.

**Aim:**

We aimed to identify facilitators and barriers for narrative elicitation and setting goals in a particular example of person‐centred care practice (University of Gothenburg Centre for Person‐centred Care, GPCC) where narrative elicitation is considered as a method of setting goals for the patient.

**Methods:**

Observation of 14 admission interviews including narrative elicitation on an internal medicine ward in Sweden where person‐centred care was implemented. Five focus group vignette‐based interviews with nurses (n = 53) were conducted to assess confirmation of the emerging themes.

**Results:**

The inductive analysis resulted in three themes about the strategies to elicit patients' narratives: (a) Preparing for narrative elicitation, (b) Lingering in the patient's narrative, and (c) Co‐creating, that is, the practitioner's and third parties' engagement in the patient's narration. Even though there were obstacles to eliciting narratives and setting lifeworld goals in a medical setting, narrative elicitation was often useful to turn general and medical goals into more specific and personal goals.

**Conclusions:**

Narrative elicitation is neither a simple transition from traditional medical history taking nor a type of structured interview. It entails skills and strategies to be practiced. On the one hand, it revitalizes ethical considerations about clinical relationship building. On the other hand, it can help patients articulate lifeworld goals that are meaningful and important for themselves.

## 1 INTRODUCTION

Revitalized interest in narrative in health care has envisaged a paradigmatic change that would reinvigorate the art of medicine^1^, ^2^ and encourage health‐care professionals to think beyond the biomedical realm. Narrative‐based approaches aim to embrace idiosyncratic experiences of illness,^3^, ^4^ enhance clinical dialogue^5^, ^6^ and empower people with medical conditions vis‐à‐vis the dominance of biomedical knowledge and language.^3^, ^4^, ^5^, ^6^, ^7^, ^8^, ^9^, ^10^, ^11^, ^12^

Some recent models of patient and person‐centred care have considered narrative as a way to attend to the person behind the patient.^12^, ^13^, ^14^ In other words, the patient is more than a diagnosis and a passive recipient of health care. Alongside the efforts to improve health‐care professionals' narrative skills and competence,^15^ some recent frameworks of person‐centred care (hereafter PCC)^13^, ^14^, ^16^, ^17^ have therefore promulgated narrative elicitation as a means to gain better understanding of the person behind the patient.

There is a wide range of definitions and practices claiming to be person‐centred.^18^, ^19^, ^20^ It is mainly construed as an overall change in health‐care organization and ethics, with less consideration for practice implications.^21^ The selected case in this study (University of Gothenburg Centre for Person‐Centred Care, GPCC) combines an ethical approach which acknowledges the capacities of the person and three routines guiding health professionals, that is, eliciting the patient's narrative, partnership and documentation. It is a particular example that interweaves a narrative‐based approach with person‐centredness. It encourages health professionals to attend to the patient as a person with capabilities, resources and a narrative to relate.^13^, ^14^, ^17^ Narrative elicitation consists of asking questions beyond the diagnostic workup, guiding the person to grasp and relate their wishes and capabilities, and probing their accounts into shape so that the patients set their own goals. It is suggested as a method for health professionals to acknowledge patients' experiences of illness and give patients space to bring their resources and goals. The underlying understanding is that narrative elicitation leads these goals to be less biomedical and technical, yet more meaningful for the persons and their lifeworld, that is, the ways in which they perceive and make sense of their illness in the context of their everyday lives.^22^, ^23^ GPCC's emphasis on narrative elicitation as a new ‘routine’ has aimed to interrupt the dominance of biomedical language^24^ and empower patients as important actors in health‐care delivery.

Yet, narrative elicitation requires organizational, technical and attitudinal changes.^6^, ^11^, ^12^ Narrative elicitation is more dynamic and unpredictable than structured ways of taking a history. There are no strict guidelines that always work, hence professionals need to develop individual and collective strategies to perform it. How narrative elicitation is used in person‐centred care practice and how it can reshape clinical routines should be further observed, examined and documented to elucidate facilitators and barriers in the process. We also need to trace whether and in which ways narrative elicitation helps patients to bring their capabilities and set goals in clinical communication. It is against this backdrop that we propose close examination of narrative elicitation as it unfolds in one ethically driven and evidence‐based practice of PCC.

The aim of this study is to identify strategies and barriers in narrative elicitation and to discuss the relationship between narrative elicitation and setting lifeworld goals. We will argue that narrative elicitation is not always straightforward in practice, and thus entails strategies to overcome the practical and professional challenges.

## 2 METHODS

### 2.1 Research design

Our previous study about the implementation of the GPCC has shown that translating PCC into practice requires contextual, sometimes contested, and often creative adaptation.^16^, ^17^, ^25^, ^26^, ^27^One commonly contested issue has been narrative elicitation, which is often complicated by divergent understandings of narrative, time restraints and established ways of clinical communication. Drawing upon the insights of this study, we designed an observational and focus group study to explore narrative elicitation as it unfolds in practice. Ulin et al^28^ ^,p. e25^ suggested that it was useful to identify ‘each patient's motivation and resources from the patient narrative already at admission’. We thus selected an internal medicine ward specialized in cardiology in a Swedish hospital where registered nurses (RN) designed and used a specific admission interview form to elicit narratives.^16^This selection was made to enable in‐depth exploration of the process in one particular setting, rather than to generalize the findings to other contexts having different characteristics and practices. The form developed on the ward, alongside questions about the patient's medical and psychosocial condition, includes open‐ended questions such as ‘would you like to tell (me/us) why you came here?’, ‘what would you like to return to?’, ‘what are your goals?’ and ‘what makes you happy?’ The RNs are expected to elicit the narrative and complete the form simultaneously. The RNs had received seminars about PCC but they were not specifically trained in narrative elicitation techniques. The absence of an established way of eliciting narratives justified an explorative study about the different strategies that the RNs develop in situ. RNs were willing to reflect upon their skills and this led to five focus group interviews with registered and assistant nurses working on the ward.

### 2.2 Data collection

The observation study took 13 days of ethnographic fieldwork^29^ over 3 months in 2017. Two ward managers and two RNs experienced in PCC facilitated access to the ward for ÖN. ÖN, trained in ethnographic methods, was on site during one whole shift each day of fieldwork. Each day of fieldwork, ÖN participated in the staff meeting before the start of the shift, and informed people about the study and his presence. As all RNs were expected to conduct admission interviews, they were first asked if they agreed to be observed by the researcher. Then, when a new patient was admitted, ÖN approached each RN in charge of the interview if he could observe it. All participating RNs consented to be observed. Then both ÖN and the RN informed the patient and asked for consent for ÖN's presence and observation during the interview. Two patients did not want to participate in the study and the researcher did not observe these admission interviews. The study was approved by the Regional Ethical Board of Gothenburg, Sweden. All participant nurses and patients were provided written and verbal information about the study and gave their consent to participate. ÖN observed 14 admission interviews. He also observed the preparation for two admission interviews without participating in the interviews themselves because one was an emergency case and patient consent could not be requested, and the second was an infection case where the patient was isolated. He wrote down notes during observation periods and elaborated these notes after the observation. He had short conversations about the admission interview with the nurse and the patient afterwards when this was possible.

Vignettes were developed to stimulate focus group discussions and to refine the findings of the observational study. The RNs were eager to discuss his observations with the researcher. ÖN wrote eight vignettes illustrating some challenges and facilitators to narrative elicitation; these were hypothetical but inspired by his observations. The research team and two RNs commented and refined the vignettes, which AW checked for medical accuracy. These were used for five focus group interviews with registered and assistant nurses (n = 53), lasting 75 minutes on average. Three vignettes were selected and introduced in each group by ÖN and AW. Nurses were asked what they thought was successful in terms of narrative elicitation in the vignette and what they would do if they were in the place of the nurse. These focus‐group discussions were audio‐recorded and transcribed. As these focus group discussions took place during staff meetings, some nurses had to leave in the middle of the discussion for other duties.

### 2.3 Data analysis

The research design led to two different sets of data. The first set consisted of the observation notes^29^ of admission interviews. These included the minutiae of admission interviews, the details of nurses' attitudes, formulation of questions, turn‐taking in conversation as well as patients' reactions, how and what type of goals were recorded, the length of the interview, and challenges during the interaction. The notes for each case were written down by ÖN, and circulated to the other team members. Team members asked ÖN questions to clarify certain details and to interrogate his interpretations. Then, ÖN coded and analysed thematically the field notes. Three themes emerged from inductive analysis.^30^ Subsequently, patients' documented goals were categorized depending on their content and nature. The research team met regularly face‐to‐face and online to discuss ÖN's ongoing analysis.

The second set of data was generated in the focus group discussions. The emerging themes from analysis informed the preparation of vignettes, where the researchers incorporated the identified strategies into scenarios. The use of vignettes aimed both to integrate multiple methods and to validate the themes through informant feedback. This feedback is a pivotal triangulation technique in qualitative research^31^ and was used to minimize the single observer's biases. For instance, the researcher was not a health professional himself and observed the professionals' use of personal information and included this theme in one scenario. This theme was elaborated after the participants' feedback.

The two sets of data were used in data analysis. For the themes for which informant feedback was positive, the analysis of field notes was used. For the presentation of contested themes, data from the focus groups were included to offset the researcher's subjectivity and to give voice to informants' objections.

## 3 RESULTS

In this section, first, we will present three main strategies for eliciting the patient's narrative. The first theme was preparing the interview, that is, what nurses do before the admission interview. The second concerns what happens during the admission interview to linger in the patient's narrative. The third was about the co‐creation of narrative via joint interviews or self‐disclosure Table 1. Second, we will elaborate on the nature of goals in relation to strategies Table 2.

**Table 1:** Strategies for narrative elicitation

Preparing for narrative elicitation	Lingering in the patient's narrative	Co‐creating narrative
Reading medical records	Active listening	Joint interview
Communication with colleagues	Using silence	Self‐disclosure
Division of labour	Changing the frequency, pace and order of questions	
Timing	Follow‐up questions	
Environment		

**Table 2:** Strategies and barriers to narrative elicitation

Case	Duration	Description	Strategies for narrative elicitation	Barriers to narrative elicitation	PCC goal
1	‐	Patient was not conscious		Case of emergency	Not elicited at that time
2	56 mins	Arrival from the emergency room, woman (50s), early retirement, dyspnoea, chest pain, fatigue, sleep problems, anxiety	Reading medical records Timing Environment Follow‐up question Active listening Joint interview Self‐disclosure		Being able to take care of her garden/flowers and household chores
3	11 mins	Arrival from another ward, man (50s), migrant, homeless, alcohol problems, cannot speak Swedish	Reading medical records	Language barrier	Not elicited at that time
4	12 mins	Arrival from the emergency, woman (40s), history of heart attack, unemployed	Reading medical records	Failed timing Lack of RN's active listening	I want to work If not, to get sick leave
5		Arrival from the emergency room, man (60s)		Case of infection, not observed (patient isolated)	
6	13 mins	Arrival from another ward, woman (80s), dyspnoea, chronic obstructive pulmonary disease (COPD)		Failed timing Lack of RN's active listening	Be healthy
7	27 mins	Arrival from the emergency room, man (50s), chest pain, work‐related previous operation			Not elicited
8	43 mins	Arrival from the emergency room. Woman (50s). Myocarditis	Reading medical records Timing Environment Active listening Using silence Circular questions Self‐disclosure		To go back to work and exercises, without being breathless
9	23 mins	Arrival from the emergency room, man (late 80s), dyspnoea, stress		Failed timing Lack of RN's active listening Environment (in the corridor)	Be healthy
10	37 mins	Arrival from the emergency, woman (late 80s), mild dementia, dizziness	Reading medical records Follow‐up questions Joint interview		Return to the elderly care home, walk and be with friends
11	67 mins	Arrival from the emergency, man (late 80s), chest pain, recent pace‐maker operation	Reading medical records Timing Environment Active listening Using silence Follow‐up questions Self‐disclosure		Restart his hobby: model ship building To be able to participate in his birthday celebration in the hospice deliberately scheduled 4 d later
12	25 mins	Arrival from the emergency room, man (50s), migration background, early retirement, chest pain		Failed timing, Lack of RN's active listening	He will think about it
13	23 mins	Arrival from the emergency, woman (late 80s), migration background, fluent in Swedish, dyspnoea			Get help for breathlessness
14	60 mins	Arrival from the emergency room, man (40s), newly arrived asylum seeker, arrhythmia	Reading medical records Timing Environment Active listening Using silence Circular questions		Want to know what it is
15	19 mins	Arrival from the emergency room, man (40s), chest pain, anxiety	Lack of active listening	Lack of RN's active listening	Go home as soon as possible
16	27 mins	Arrival from the emergency room, woman (70s), chest pain, fatigue, pace‐maker related anxieties, chest pain	Timing Environment Active Listening Using silence Circular questions		Taking walks with her friends

### 3.1 Strategies of narrative elicitation

#### 3.1.1 Preparing for narrative elicitation

Narrative elicitation is a difficult task on the specialized wards where patients are generally admitted for specific and relatively short‐term interventions. The turnover of patients is also high. Traditionally, it is common for nurses to read patients' records before the admission interview. Some nurses in this study explicitly used prior information in order not to repeat the questions that the patient had already answered in the emergency room. This also enabled them to prepare the admission interview, not in a structured way, but by having some intuition about the person. Reading the patient's medical history helped imagining and predicting the social, personal story of the patient. It was often guesswork based on sparse information like the patient's residence, profession and family members. For instance, the information from the medical records helped the nurse to ask ice‐breaking questions to the patient about her exercises:

While carefully examining the documents on the screen N8 informs me that the patient that we will admit is a woman in her early 50s [and some other personal information]. She has reported chest pain at the emergency room. She is an athletic person, she regularly exercises. [Field note, case 8]

In order to create time for an interview without unnecessary interruptions, some nurses informed their co‐workers. This involved clear communication about who would conduct the interview and when. The high turnover of patients and variety of tasks entailed strategic planning of tasks and division of labour. In several cases, nurses first took care of other time‐bound tasks (such as distributing medicines). Similarly, some tried not to commence admission interviews just before lunch or the end of their shift.

N11 is informed about a new admission. N11 starts reading the patient's medical reports and filling some of the yes/no questions on the form. N11 decides to distribute other patients' medication before the patient arrives so that she is not disturbed during the interview. [Field note, case 10]

Providing privacy for the admission interview was not always an easy task because of the shortage of available beds and rooms hosting two to four patients at a time. Some practices like closing doors and inviting other patients to the coffee room when possible seemed to be basic but useful in making patients comfortable.

The patient is sitting on a stretcher in the corridor since they are about to discharge a patient and there are no available beds. He is older old and has some hearing problems. N10 conducts the admission interview in the corridor, she kneels down, starts asking questions aloud. There is no privacy, other nurses passing by and greeting N10. [Field note, case 9]

#### 3.1.2 Lingering in the patient's narrative

Active listening is about skilfully navigating conversation and silence without interruptions during face‐to‐face communication.^12^, ^32^ Narrative elicitation required nurses to be more attentive to the interaction and to provide patients with the time and attention they needed. Taking a seat, having a calm posture and keeping eye contact were some common strategies. As some patients were not familiar with PCC, they were unfamiliar with narrative‐inducing questions (such as ‘*Could you tell me what happened this morning before you came here?'*).

I am with N15, a male nurse in his early late 30s. He is conducting an interview with a female patient in her 70s who had a pace‐maker fitted recently. N15: ‘How are you?’ P: ‘I am not well’. (She starts talking about a medicine that she has had for 4 months, and she had diarrhoea and lost 6 kilos). N15: ‘What have you done today before you came here?’ P: ‘I had a heart throbbing. It started Monday but it got worse today’. N15: ‘Did you feel better when you came here?’ P: ‘Yes, it feels safe’. The patient starts talking about her daily life, how this medicine has affected her life, and the difficulties of having a pace‐maker. [Field note, case 16]

In the above case, the nurse's attentive and calm attitude helped the patient tell her story without interruption. Unlike this patient, many often tended to give affirmative and short responses rather than long accounts. Not rushing the conversation, being silent and waiting, and not posing a new question if the patient needed time to remember or formulate an answer were useful to encourage patients to give longer accounts.

Narrative elicitation was not always compatible with medical questions on the form. Nurses developed two strategies to overcome this difficulty. Some preferred slightly changing the frequency, pace and order of medical questions and avoided mechanically juxtaposing questions. Others tried to ask short follow‐up questions during the narrative elicitation. For instance, in one case (2), when the patient told about her recent journey to another country, the nurse asked if she visited a doctor there, received the information and filled the form without interrupting the narrative flow.

One way to linger in the patient's narrative was asking questions exploring the same topic further with follow‐up questions.^11^ This included repeating some details of the story, asking follow‐up questions if necessary, alluding to significant events in the narrative (such as a recent heart attack or loss of appetite), helping patients connect these to potential wishes, plans and goals (such as attending a dinner, travelling, starting or commencing a new hobby).

#### 3.1.3 Co‐creating narrative

There were two ways in which narratives were created in dialogue: firstly through self‐disclosure and secondly through joint interviews, that is, interviews including third parties as facilitators or information resources in communication.^17^, ^33^ Self‐disclosure as provision of information about the clinician's life outside the admission interview^34^ depended on the clinical encounter. Even though there were examples of skilful and well‐balanced self‐disclosure in the observed cases, many nurses reported ambivalent attitudes to self‐disclosure during focus group interviews. On the one hand, they considered self‐disclosure as relationship building that encourages patients to open up more comfortably and articulate their goals. On the other hand, there was a fear that it might over‐personalize the conversation expected to be formal.

I don't know, I usually… maybe it is not right but I usually don't talk about myself a lot, my own family situation. I can talk a bit superficially like I rode a bicycle to work and so. I try not to take my personality in. […] But, on the other hand, I ask him personal questions. (N1, Focus group 1)

This ambivalence also crystallized the paradox of partnership in person‐centredness: patients are encouraged to bring their personal accounts to the clinical dialogue, whilst detachment as a prevalent professional ideal leads some nurses to make economic use of their personal stories.

Nurses often elicited narratives in the presence of family members, close friends and partners. These joint interviews were another method used to create a narrative. Family members and friends were often seen and treated as resource for narrative elicitation, especially in the cases where the patient had memory problems (eg case 10) but also when facing language barriers (eg case 2).

The patient looks a bit dizzy and she walks with the help of her son. N11 greets them and invites them to a room for two. I introduce myself and we get consent for my presence. N11 starts talking with the son, asks about her medicine. The patient is calm but a bit distracted. N11 is confident and cheerful; she makes some jokes to the patient. N11 decides to listen to the patient's heart, her son helps with instructions, and he makes his mother breathe deeply and helps her answering the questions. [Field note, case 10]

Many nurses also reported in focus group interviews that family members could be useful as interpreters despite some ethical dilemmas involved.

This is something that one notices very quickly. [Relatives] Taking over or [being] a resource [in the interview] if they complement [what the patient says], reminding us about the balance there. (N1, Focus group 1)

Yet, given the ethical stance to attend to ‘the person’ and hear their own goals, some were hesitant to collaborate with third parties in eliciting the narrative.

Relatives can take very much space (…). It was really good once that we were two because the patient's biggest problem was impotence and he did not want to talk about it with his wife. He thought that it was very hard. (N2, Focus group 1)

### 3.2 Goal setting

#### 3.2.1 Obstacles to eliciting narrative and setting lifeworld goals

A central motivation for eliciting narratives is to enable patients to bring their lifeworld goals into care planning. Nevertheless, this was not clear in practice for three reasons. First, given the specialty of the ward, some patients had urgent symptoms and they were not accustomed to narrative approach in the first encounter.

First of all, patients perhaps don't understand that we ask them what they want. I think that one is not very used to it in traditional health‐care situation. (Imitating) ‘I am the patient, you will tell me what I should do, what it is this about?’ One does not expect the question: what do you want? What are your wishes, how do you see this situation? […] I think that our new way of working is not established for patients. (N9, Focus group 1)

As some patients had long‐standing illnesses, they were also used to talking and asking questions in medical language. This posed an obstacle to address other concerns and talk about lifeworld goals.

Secondly, the relationship between narrative elicitation and setting lifeworld goals was not self‐evident for many nurses. In some cases, patients were happy and eager to tell their stories, yet there was need for interpretation of these accounts to articulate a lifeworld goal. When patients gave longer accounts of their illness experiences, daily lives, family relations and work situations, there emerged several goals. Narrative elicitation did not necessarily lead to specific lifeworld goals. Some nurses thus endeavoured to bridge this gap by referring strategically to specific activities that patients named, for instance, physical and leisure time activities. The third obstacle was the lack of continuity in setting lifeworld goals. In some cases, it seemed challenging and beyond their professional competence and boundaries to help patients to work for the stated lifeworld goals. For instance, in one case (4), the patient spoke about her prolonged unemployment and the nurse conducting the interview was confused about setting the goal, which would go beyond what he could achieve within his professional boundaries.

Despite these obstacles, many nurses reported in the focus groups that narrative elicitation enabled them to be more attentive to the person. Many were positive about the relationship‐building aspect of narrative elicitation. Yet, there were also open criticisms about the timing of narrative elicitation in the focus group interviews. Some argued that narrative elicitation and goal setting should not be confined to the admission interview but spread over time.

If I were a patient, I would block personal things of my private life, what I do in the future. I would think that it is not directly about care but by slowly building a relationship, which we don't do the first 11 minutes. (N22, Focus group 2)

#### 3.2.2 Generic vs specific goals

The patients' goals in this study can be classified into two groups. The first group consisted of general goals like ‘being healthy’ (cases 6 and 9) or specific medical goals like ‘getting help for breathlessness’ (case 13). The second group consisted of goals that are specific to the patient's everyday life. There were often challenges about conducting the interview properly and listening to the narrative. Regardless of how engaged they became in narrative elicitation, many patients stated the goal of ‘being healthy’ as an initial answer to the question ‘*What would you like to return to*?’ (Case 10). Some nurses were aware of this tendency and therefore endeavoured to help patients identify more specific goals with follow‐up questions (such as ‘*Ok, what would you like to do when you are healthy again?’* (Case 4), ‘*Would you like to be able to spend more time in your garden when you go home?’* (Case 2)). They also referred to patients’ accounts and some activities that sounded meaningful for the patient (such as ‘*You told me that you wanted to spare more time for model ship building. Is it still important for you?’* (Case 11)). These specific questions led to more specific lifeworld goals such as ‘restart his model ship building’.

As the Table 2 illustrates, there is no simple correlation between the strategies used in narrative elicitation and the specificity of the goals. In case 14, for instance, even if the nurse combined different strategies like active listening and using follow‐up questions, it was not possible to set a specific lifeworld goal since the patient was surprised to be hospitalized for the first time and concerned about the diagnosis, whereas in case 4, the patient felt comfortable giving a narrative account and identifying a specific goal despite the nurse's bad timing and failure to listen attentively to the narrative. These two cases also show that patients have different expectations from telling their stories.

However, it is possible to point to one common pattern: the more nurses engaged in preparing, lingering in the patient's narrative and relationship building, the more successful they were in setting goals that were more specific. For instance, in cases 2, 8, 11 and 16, nurses were more successful in setting specific lifeworld goals because they deployed several strategies simultaneously and could ask relevant follow‐up questions to the initial response about ‘being healthy’.

## 4 DISCUSSION

Our findings point to the rich plurality of the strategies that professionals have been developing since the implementation of the GPCC framework in 2010. These included preparations prior to the admission interview, asking narrative‐inducing questions and listening attentively to the patients, and relationship building. Professionals’ engagement in narrative elicitation is likely to guide patients in expressing more specific lifeworld goals.

Preparations prior to the interview help avoid interruptions, repetition of the same questions or lack of concentration. Reading medical records may provide some biographical information to be used as ice‐breaking questions. Even though this strategy is observed to be useful, the high turnover of patients makes it difficult to use systematically.^35^

Patients are often allocated short consultations and health‐care professionals feel more and more pressure to be time effective and pose questions addressing medical issues.^6^, ^8^, ^11^ Asking questions beyond the medical realm not only requires time and well‐organized division of labour, but also skills of formulating narrative‐inducing questions and active listening^32^ as the patient's narrative during the admission interview emerges ‘in the context of requests, acknowledgements, expansions, and elaborations’.^36^ ^,p. 368^ Some nurses managed to elicit narratives by drawing upon their professional and personal experience but training programmes could endorse and sustain these skills.^27^

Narrative elicitation has revitalized some ethical dilemmas. Self‐disclosure^34^ and joint interviews^17^, ^33^ are observed to be two ways of co‐creating narrative, but professionals also reported their concerns about these strategies. The tension between the occasional self‐disclosure that they often unconsciously use with certain patients and the prevalent ideal of professional detachment becomes crystallized since narrative elicitation requires more attention and reciprocity in the form of acknowledging the person and participating in creation of narratives. There are also concerns about turning an admission interview into an ordinary conversation. Narrative elicitation provokes discussions about professional boundaries, relationship building in clinical communication and the limits of reciprocity.^37^ However, just as Mishler was concerned about which aspects of patients' lives are appropriate topics in medical interviews,^24^ there is also a question about which aspects of professionals' life worlds are appropriate to meet the narrative and normative requirements of a clinical interview.

The ambivalence about joint interviews is another issue. This can hark back to some criticisms to the narrative turn that was considered to emphasize ‘the isolated actor who experiences and narrates as a matter of private and privileged experience’.^38^ While for some family members or close friends were considered helpful partners in elicitation, others considered them harmful to the dialogue between the nurse and the person. This is an understandable concern in the case of family members who take control of the conversation and attempt to impose their own agendas. Having joint interviews in PCC appears somehow contradictory as if one's narrative would not be genuine if third parties were involved in narrative elicitation, but models of PCC generally need to have a more relational vision of personhood and acknowledge that the uniqueness of the person is always shaped and expressed through a web of relations.^17^Given the potential contribution of third parties in both narrative elicitation and goal setting in some observed cases, it is not a clear‐cut question. One way to acknowledge potential benefits but also harms of the third parties in narrative elicitation can be to go beyond this individualistic and dyadic understanding of narrative elicitation^17^by taking the person's web of relations critically and informatively into consideration.

This study also calls into question the relationship between eliciting narratives and setting lifeworld goals. Some patients persevere in expressing goals like ‘being healthy’. This points to some challenges that narrative‐based approaches face: many patients are not familiar with narrative elicitation in medical settings and may even prefer to focus on medical issues.

Many people have long been inclined to focus on their medical conditions during medical history taking, since their resources and capabilities are rarely taken into account in clinical communication, not only in admission interviews. Narrative elicitation is therefore a way to open this space for acknowledging their resources and capabilities. While ‘being healthy’ is a more generic goal, often renegotiated by people with chronic illnesses, ‘being able to do gardening’ is more precise and arguably more motivating to take part in care planning. To decide whether these specific goals are genuine and attainable^16^or not is beyond the scope of this article. Yet, they are arguably the result of more reciprocal and person‐centred communication.

Narrative elicitation is less structured and predictable than other forms of history taking and goal setting. Yet, however, unpredictable and difficult narrative elicitation may still contribute to person‐centredness in bringing forth what the patients consider as meaningful and important for themselves. Thus, it is more plausible to point to certain skills and strategies rather than providing a standardized set of guidelines that would always work in every health‐care setting. Our study identified strategies of narrative elicitation which were performed on a specific ward, but there is a need for further research addressing the contextual variations of the use of narrative in different settings.

This study focused on observations of narrative elicitation on a specific ward. The themes generated by the situated observation of the researcher were triangulated with continuous feedback from the nurses and focus group interviews. Different settings may present other strategies and realities depending on the context. It is also difficult to point to a particular set of strategies that always work. However, it is possible to highlight some common patterns in eliciting narratives. As is the case with all observation studies, research participants might have paid more attention to what they did and how in the presence of a researcher. They might have attempted to demonstrate best practice, but this was equally valuable for the aim of this study.

## 5 CONCLUSION

Narrative elicitation is neither a simple transition from traditional medical history taking nor a type of structured interview. It entails skills and strategies to be practiced. On the one hand, it revitalizes ethical considerations about clinical relationship building, while on the other hand, it can help patients articulate lifeworld goals that are meaningful and important for themselves.

## CONFLICT OF INTEREST

The author(s) declared no potential conflicts of interest with respect to the research, authorship and/or publication of this article.

## AUTHORS’ CONTRIBUTIONS

All authors contributed to the study design and development. ÖN conducted the observational study and drafted the manuscript. All authors were responsible for critical revision and finalising the manuscript. All authors read and approved the final manuscript.

## Data Availability

The data that support the findings of this study are available from the corresponding author upon reasonable request.
